# Candida graft arteritis after kidney transplantation: two cases and literature review

**DOI:** 10.1093/omcr/omaf172

**Published:** 2025-09-28

**Authors:** Tirlangi Praveen Kumar, Attur Ravindra Prabhu, Pothumarthy Venkata Swathi Kiran, Arun Chawla, P S Priya, Brij Mohan Kumar Singh, Nitin Gupta

**Affiliations:** Department of Infectious Disease, Kasturba Medical College, Manipal, Manipal Academy of Higher Education, Manipal, Karnataka 576104, India; Department of Nephrology, Kasturba Medical College, Manipal, Manipal Academy of Higher Education, Manipal, Karnataka 576104, India; Department of Infectious Disease, Kasturba Medical College, Manipal, Manipal Academy of Higher Education, Manipal, Karnataka 576104, India; Department of Urology, Kasturba Medical College, Manipal, Manipal Academy of Higher Education, Manipal, Karnataka 576104, India; Department of Radiology, Kasturba Medical College, Manipal, Manipal Academy of Higher Education, Manipal, Karnataka 576104, India; Department of Pathology, Kasturba Medical College, Manipal, Manipal Academy of Higher Education, Manipal, Karnataka 576104, India; Department of Infectious Disease, Kasturba Medical College, Manipal, Manipal Academy of Higher Education, Manipal, Karnataka 576104, India

**Keywords:** Candida arteritis, mycotic aneurysm, kidney transplant

## Abstract

Background: Candida arteritis of a kidney allograft represents a severe yet rare complication in transplant recipients. Its nonspecific presentation and diagnostic difficulties necessitate a high level of clinical suspicion and a multidisciplinary approach to management.

Case presentation: We report two cases of Candida arteritis in kidney transplant recipients who presented with life-threatening bleeding from the graft anastomotic site shortly after transplantation. Histopathological examination revealed fungal invasion of the arterial walls, with periodic acid-Schiff (PAS) staining demonstrating budding yeast cells and pseudo hyphae. Both patients underwent emergency graft nephrectomy and iliac vessel repair. Antifungal therapy with intravenous fluconazole was initiated. Despite these interventions, one patient succumbed to a rebleed ten days postoperatively, while the other survived but experienced graft loss.

Conclusion: This report highlights the importance of early recognition, maintenance of sterile conditions during organ transport and vigilant postoperative monitoring to minimise the occurrence of this life-threatening complication burden.

## Introduction

Candida arteritis of the kidney allograft is a rare and life-threatening complication [1]. The graft kidney can be infected in the donor, during retrieval, after retrieval (due to contaminated preservative fluid), or after it has been transplanted. In the graft, *Candida* species seed the vasculature and lead to arteritis [2]. Due to the nonspecific nature of its symptoms and the challenges in obtaining positive microbiological cultures, Candida arteritis is often diagnosed late. This can lead to significant morbidity and mortality. In this series, we report two patients with Candida graft arteritis presenting with life-threatening bleeding from the anastomotic site after kidney transplantation (Table 1).

## Case report

### Case 1

**Figure 1:**
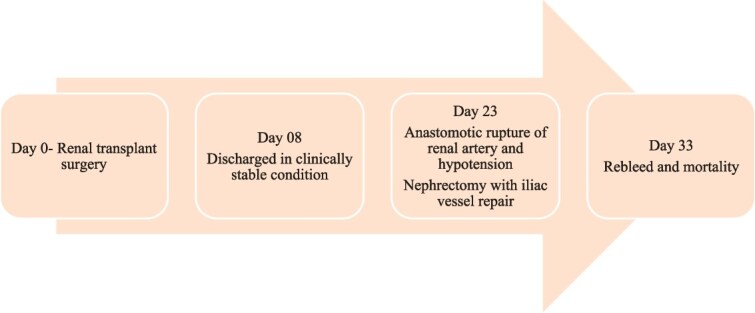
Timeline of events for patient 1 following the transplant.

A 38-year-old hypertensive male with end-stage kidney disease (ESKD) on maintenance haemodialysis underwent deceased donor kidney transplantation. Postoperatively, the patient had delayed graft function, gradually improving, reaching a nadir serum creatinine of 1.2 mg/dl at discharge. The screening Doppler conducted before discharge was normal. After fifteen days of discharge, he presented with abdominal distension, oliguria, and breathlessness. There was no history of fever or haematuria. On examination, he was pale, hypotensive and had graft tenderness. Laboratory investigations showed severe anaemia, neutrophilic leucocytosis, acute kidney injury, deranged coagulation, severe metabolic acidosis and candidemia. Contrast-enhanced Computed Tomography (CECT) identified active contrast extravasation from the graft vessel anastomosis site, leading to hemoperitoneum (Fig. 3A). The patient underwent an emergency laparotomy, which confirmed a rupture of the kidney artery at the anastomotic site accompanied by graft necrosis. A graft nephrectomy and repair of the iliac vessels were performed. Histopathological analysis, tissue culture of the graft and renal artery revealed features suggestive of candida arteritis (Fig. 4A). Despite graft removal, antifungal therapy (fluconazole), and supportive care leading to clinical improvement and successful extubation, the patient succumbed to a rebleed after ten days Figure 1.

**Table 1:** Clinical profile, laboratory features, treatment and outcome of patients with Candida graft arteritis.

Parameters	Reference range	Patient 1	Patient 2
Comorbidities		HT, hepatitis C (cured)	HT, IHD
Donor		Young male died following road traffic accident	Young female died following anaphylaxis
Induction		Basiliximab	Anti-thymocyte globulin
Maintenance chemotherapy		Tacrolimus, mycophenolate mofetil, and prednisolone	
Haemoglobin (g/dl)	13–17	6.1	4.2
Leucocyte count (cells/mcl)	4–10	7300	10 200
Platelet count (cells/mcl)	150–400	119 000	114 000
Creatinine (mg/dl)	0.7–1.2	1.3	8.89
Blood pH	7.4–7.45	7.20	7.42
Bicarbonate (mmol/l)	22–29	7.0	17.3
Blood culture		*Candida albicans*	*Candida tropicalis*
Tissue culture		*Candida albicans*	*Candida tropicalis*
Fluconazole susceptibility		Susceptible	Susceptible
Histopathology		Kidney artery studded with budding yeast cells and pseudohyphae	
Antifungals		Fluconazole Intravenous 400 mg/day	Fluconazole Intravenous 400 mg/day for 2 weeks followed by Oral fluconazole 400 mg/day
Outcome		Death	Alive (graft loss)

Abbreviation: HT- hypertension, IHD- Ischaemic heart disease

**Figure 2:**
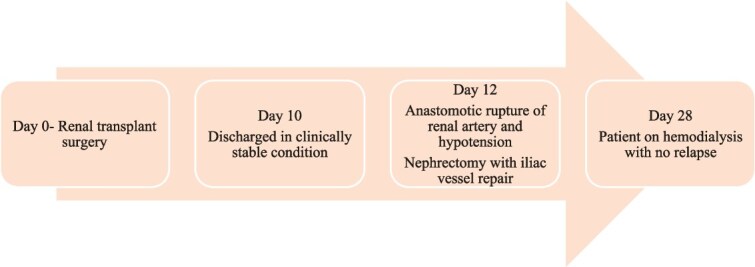
Timeline of events for patient 2 following the transplant.

### Case 2

**Figure 3:**
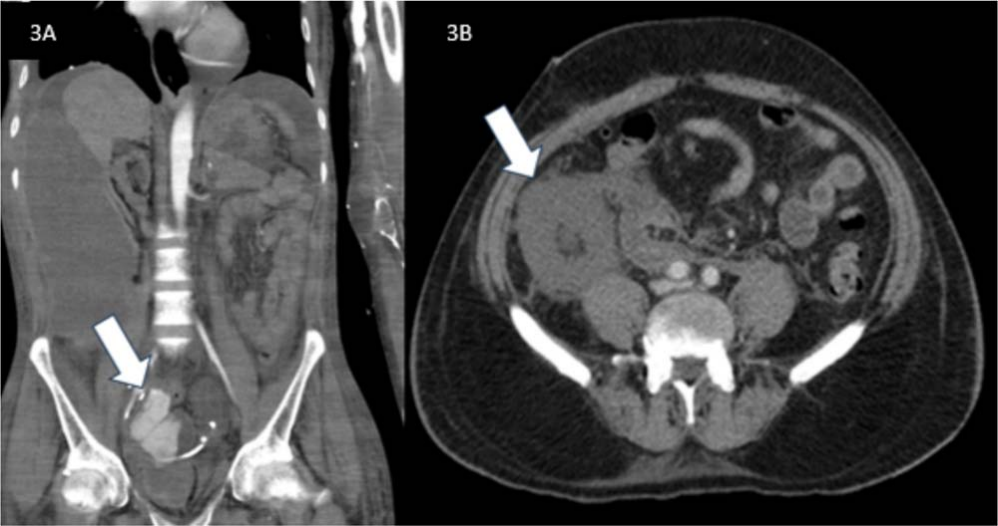
Computed tomography of the abdomen with panel 1A showing a large right abdominal hyperdense collection with active arterial extravasation (arrow) from graft anastomotic site, compressing adjacent organs in case 1 and panel 1B showing grafted kidney with no enhancement (arrow) of the parenchyma or vascular pedicle, indicating likely graft infarction in case 2.

**Figure 4:**
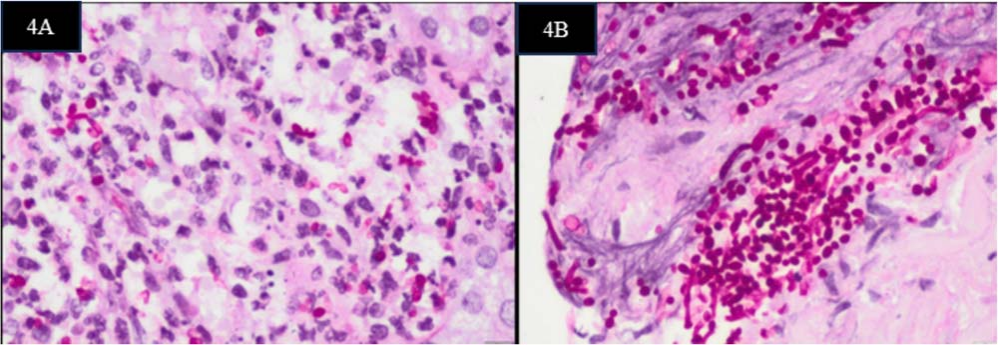
Histopathological examination of the grafted kidneys on PAS showing yeast cells inside the injured glomeruli in Case1 (A) and along the arterial wall in case 2 (B).

A 51-year-old male with ESKD on maintenance haemodialysis for four years received deceased donor kidney transplantation. He had delayed graft function, requiring one session of haemodialysis after the transplant, and an allograft kidney biopsy done on the third day was reported as acute tubular necrosis with no evidence of rejection or infection. He was discharged after he went into a diuretic phase on the tenth day post-transplant with a serum creatinine level of 7.3 mg/dl and a haemoglobin of 7.8 gram/dl. The screening Doppler conducted before discharge was normal. He returned two days later with lower abdominal discomfort, postural symptoms and hypotension. He had severe anaemia, haemoglobin 4.8 gram/dl, thrombocytopenia, neutrophilic leucocytosis, and blood cultures later returned as candidemia (*Candida tropicalis*). Ultrasound and Doppler imaging showed a peri graft collection with absent perfusion. CECT abdomen confirmed features of absent graft perfusion and retroperitoneal hematoma (Fig. 3B). Immediate exploratory laparotomy, graft nephrectomy, and iliac vessel repair were done. Histopathological examination of the excised graft revealed widespread infarction with extensive yeast invasion in the interstitium and the renal vessel walls (Fig. 4B). A repeat blood culture was sterile three days after initiating fluconazole. The patient was restarted on haemodialysis and continued on fluconazole (planned to continue for 3 months) Figure 2.

## Discussion

Candida arteritis is a rare but severe complication following kidney transplantation. Although its incidence is low, it holds considerable clinical significance due to its aggressive progression, resulting in graft loss and mortality [3]. These outcomes are further compounded by the challenges of achieving an early diagnosis as they often present without fever and/or negative blood cultures [3]. Clinically, Candida arteritis commonly presents as a direct manifestation of arterial dehiscence, causing haemorrhagic shock without preceding signs or symptoms [4–6]. Both patients presented within a few days of discharge with shock. As observed in our patients, the anastomotic site is the common site of arterial dehiscence. However, mid-arterial and hilar rupture of kidney arteries is reported [7]. Candida arteritis can also present as a kidney artery aneurysm, which may or may not be associated with graft dysfunction [8, 9]. In rare cases, embolic complications such as septic arthritis may also occur as presenting features [10].

At our centre, we routinely perform Doppler evaluations of graft perfusion three times a week in cases of delayed graft function. In both patients, perfusion was consistently found to be adequate, with no evidence of aneurysms noted until discharge. However, both patients later presented with anastomotic dehiscence and life-threatening bleeding, highlighting the sudden and unpredictable onset of Candida arteritis.

Imaging studies play a critical role in identifying vascular involvement. Contrast extravasation at the anastomotic site, indicative of arterial rupture, was noted in Case 1. At the same time, Case 2 showed an absence of the vascular pedicle and parenchymal enhancement, suggestive of graft infarction. A definitive diagnosis of Candida arteritis requires microbiological or histopathological confirmation, with fungal invasion of the arterial wall as a disease hallmark.

Candida infection can originate from an infected donor, during organ retrieval, or through contamination of the preservation fluid [2, 11]. Risk factors for Candida arteritis can be categorised as donor-related, such as a history of abdominal trauma, non-heart-beating donors, and prolonged ICU stays with antibiotic treatment; surgery-related, including gut leakage during organ retrieval, multi-organ donation, and contamination of the preservation fluid; or recipient-related, such as urogenital colonisation and immunosuppressive therapy [12, 13].

Routine preservative fluid cultures can help guide prophylactic antifungal treatment. However, not all patients with positive preservative fluid cultures develop Candida arteritis, and similarly, negative preservative fluid cultures do not eliminate the risk of Candida arteritis [2, 11, 14, 15]. The efficacy of postoperative prophylaxis is inconsistent, partly due to the delayed positivity of Candida cultures. Interestingly, in some cases, preservation fluid cultures that initially grow coliforms have been associated with the subsequent development of Candida arteritis [6, 11]. Additionally, due to bacterial overgrowth, Candida growth may be missed when fungal culture bottles are not used. The use of fungal culture bottles and advanced rapid molecular diagnostic techniques can reduce the time required to identify contamination in preservation fluids, thereby optimising prophylaxis [16, 17]. When preservation fluid cultures are positive, antifungal prophylaxis and close radiological monitoring for aneurysms might be helpful. Some centres also advocate routine second-look surgery to assess vascular integrity at the anastomotic site [15]. Our patients received kidneys from multi-organ donors; there was no documented evidence of gut leakage during organ retrieval.

The timing of diagnosis and intervention strongly influences the prognosis of Candida arteritis. Early surgical resection combined with targeted antifungal therapy significantly improves survival rates and reduces the risk of life-threatening complications such as aneurysm rupture. Conversely, delays in diagnosis often result in poor outcomes, including high mortality rates [1, 11, 12, 18, 19].

The management of Candida arteritis involves surgery and prolonged antifungal therapy. Surgical options include resection of the infected artery with direct repair, resection of the aneurysm with defect repair using graft material, and resection of the aneurysm with ligation of the external iliac artery followed by femoral-femoral bypass. In many patients, graft removal is required due to the risk of rebleeding, although occasional cases of graft salvage have been reported in patients presenting with aneurysms without anastomotic dehiscence [5, 6, 9, 19]. The choice of surgical procedure depends on suspicion of Candida arteritis, the extent of involvement, and the involvement of the external iliac artery. While external iliac artery ligation with bypass is considered the gold standard, direct surgical repair is often performed in patients who are not suspected of having Candida arteritis at the time of surgery, which may be associated with a high risk of rebleeding [4, 7, 20]. Our patients were suspected to have mycotic anastomotic rupture at the time of anastomotic dehiscence, and direct iliac repair was performed. However, direct iliac repair may be associated with a risk of rebleeding, as seen in our Case 1.

*Candida albicans* is the most common Candida species associated with arteritis. Its phospholipase activity and stimulation of prostaglandin production contribute to endothelial injury [21, 22]. Antifungal therapy is selected based on the organism’s susceptibility profile. Since the optimal treatment duration is unclear, patients generally require prolonged antifungal therapy with careful monitoring to detect any recurrence of aneurysms.

## Conclusion

In kidney transplantation, allograft Candida arteritis presents a challenging prognosis, with high rates of mortality and morbidity. In patients presenting with hypotension or retroperitoneal bleeding early in the post-transplant period, clinicians should strongly consider Candida arteritis in the differential diagnosis, even when blood cultures are negative. This report highlights the importance of early recognition, maintenance of sterile conditions during organ transport and vigilant postoperative monitoring and antifungal prophylaxis to minimise the occurrence of this life-threatening complication burden.
